# Associations of Physical Activity and Television Viewing With Depressive Symptoms of the European Adults

**DOI:** 10.3389/fpubh.2021.799870

**Published:** 2022-01-12

**Authors:** João Santos, Andreas Ihle, Miguel Peralta, Christophe Domingos, Élvio R. Gouveia, Gerson Ferrari, André Werneck, Filipe Rodrigues, Adilson Marques

**Affiliations:** ^1^Faculty of Human Kinetics, University of Lisbon, Lisbon, Portugal; ^2^Center for the Interdisciplinary Study of Gerontology and Vulnerability, University of Geneva, Geneva, Switzerland; ^3^Department of Psychology, University of Geneva, Geneva, Switzerland; ^4^Swiss National Centre of Competence in Research LIVES—Overcoming Vulnerability: Life Course Perspectives, Lausanne, Switzerland; ^5^CIPER, Faculty of Human Kinetics, University of Lisbon, Lisbon, Portugal; ^6^ISAMB, University of Lisbon, Lisbon, Portugal; ^7^Life Quality Research Centre, Rio Maior, Portugal; ^8^Departamento de Educação Física e Desporto, Universidade da Madeira, Funchal, Portugal; ^9^Interactive Technologies Institute, LARSyS, Funchal, Portugal; ^10^Universidad de Santiago de Chile (USACH), Escuela de Ciencias de la Actividad Física, el Deporte y la Salud, Santiago, Chile; ^11^Laboratorio de Rendimiento Humano, Grupo de Estudio en Educación, Actividad Física y Salud (GEEAFyS), Universidad Católica del Maule, Talca, Chile; ^12^School of Public Health, Universidade de São Paulo, São Paulo, Brazil; ^13^ESECS – Polytechnic of Leiria, Leiria, Portugal

**Keywords:** exercise, quantitative study, sedentary living, depression, mental health

## Abstract

**Background:** While mentally passive sedentary behavior such as television viewing (TV) is often related with depressive symptoms, some research shows that physical activity (PA) may attenuate this association. Thus, this study aimed to examine the associations between TV, PA, and depressive symptoms, considering sociodemographic covariates.

**Methods:** A sample of 29,285 adults (13,943 men; 15,342 women) with a mean age of 50.9 ± 17.4 years (50.6 ± 17.3 men; 51.1 ± 17.5 women) from the European Social Survey agreed to be respondents for this study. Data for sociodemographic variables, TV watching, PA, and depressive symptoms were self-reported. Different statistical procedures were conducted to provide evidence for the association between study variables. ANCOVA was used to analyze the association between TV watching and depressive symptoms. Linear regression analysis was conducted to analyze the association between PA and depressive symptoms. General Linear Model was performed to analyze the association of TV watching and on depressive symptoms, controlling for PA.

**Results:** European adults who responded watching more than 2 h per day showed higher scores for depressive symptoms. Higher participation in PA was negatively and significantly associated with depressive symptoms in men (β = −0.15, 95% CI: −0.18, −0.13), and women (β = −0.23, 95% CI: −0.26, −0.21). Men spending 1–2 h/day TV watching and engaging in PA ≥ 5 days/week presented the lowest scores on depressive symptoms. The lowest scores on depressive symptoms was observed in women engaging 2–4 days/week in PA and spending <1 h/day in TV watching.

**Conclusions:** More time spent in TV watching is related with increased scores on depressive symptoms. However, regular PA participation can weaken this association.

## Introduction

Depression has been described as one of the most prevalent mental disorders among adults for the last two decades (1), and is one of the leading causes of disability worldwide (2). Despite depression affecting people of different ages and sexes, it is nearly twice as common in women than in men (1). The difference in the prevalence of depression between men and women is linked to biological and psychological differences in susceptibility and environmental factors operating in both microlevel and macrolevel (3). For instance, women undergo internal endocrine processes associated with the reproductive cycle, increasing the risk of depression during reproductive age (4). Furthermore, depression is associated with increased vulnerability, mortality and reduced life expectancy (5), mainly due to a higher risk for developing cardiovascular diseases (6). Therefore, it is expected an increased economic burden due to treatment costs and productivity loss (7). Thus, new strategies to prevent depression is paramount for public health authorities.

The symptoms associated with depression affect one's ability to function at work and to deal with daily life events. Thus, depressive symptoms mark an impact in the quality of life and are indicative of clinical depression. Symptoms such as: psychosocial incapacity; reduced productivity in work and day-to-day activities; higher risk of work absenteeism; depressed humor; changes in appetite; fatigue or loss of energy; reduced ability to concentrate, think and make decisions; lack of interest or pleasure are some of the symptoms of possible depression.

Among the protective factors for depressive symptoms, physical activity (PA) has been associated as a major contributor to mental health (8–10). Additionally, regular physical exercise reduces the incidence and severity of health problems and chronic diseases, and increases quality of life, thus making it an important constituent of health promotion (11). The scientific evidence continues to build as physical exercise is also associated with physical and mental benefits that can start accumulating with small frequency (e.g., 2 times per week) every week (12). PA related to depressive symptomatology, there is evidence that individuals with higher levels of PA had a 17% lower risk for depression when compared with those with lower PA (8).

Sedentary behavior (SB) is defined as any behavior characterized by an energy expenditure ≤ 1.5 metabolic equivalents such as sitting, lying, television watching (13). Initial effort to better understand SB levels has led to growing research interest in recent years related to its associated health risk. Current evidence has showed SB as a risk factor linked to increased depressive symptoms (14). Previous research has shown that mentally-passive behavior, such as watching TV, can be associated with mental illness (14–16). Specifically, more time spent on TV viewing is related to increased levels of depressive symptoms. Research suggests that spending longer periods of time watching TV was associated with a 13% increased risk of depression (17).

Methodological challenges to the research of physical activity and TV viewing associated with depressive symptoms include the need for large and heterogeneous samples that can build robust evidence to identify patterns. This might be particularly difficult if we consider only one region or culture (e.g., Portugal) since individuality and other sociodemographic variables may display some moderating role (e.g., age and sex). To the best of our knowledge, a proper investigation of the relationships between PA, SB, and depressive symptoms, considering the moderating role of sociodemographic variables, has only begun recently (10, 18). In addition, most of the previous studies have focused on only one country or culture, limiting interpretation of the results. Most of the studies have partially examined the associations between these variables suggesting the need to explore in detail the different relationships between PA, SB, and depressive symptoms, as a mean to provide solid evidence for researchers and public health agents to reverse current trends of increased physical inactivity, increase SB engagement, and increased levels of depressive symptoms in the European population (19, 20).

While previous studies have shown that PA can reduce the detrimental effects of TV watching on a variety of psychological distress indicators (16, 21), there is still a gap on how these associations are similar among different cultures. Considering that clinical depression is expected to increase over the following years, and since TV viewing and PA are associated with depressive symptoms (8, 17), this study aimed to: (1) analyze the independent association between time spent TV watching and depressive symptoms, as well as, PA participation and depressive symptoms; and (2) investigate the potential protective effect of PA in the relationship between TV watching and depressive symptoms in a large sample of European adults. The exploratory hypothesis was that TV watching would have a positive and significant association with depressive symptoms. On the other hand, PA would display a negative and significant association with depressive symptoms. Last, PA would attenuate the positive association between TV viewing and depressive symptoms, considering the moderating role of age and gender. These assumptions are based on limited evidence.

## Methods

### Participants and Procedures

Data was obtained from the European Social Survey (22), which included 20 European countries (i.e., Austria, Belgium, Czech Republic, Denmark, Estonia, Finland, France, Germany, Hungary, Ireland, Lithuania, Netherlands, Norway, Poland, Portugal, Slovenia, Spain, Sweden, Switzerland, and United Kingdom). The European Social Survey (ESS) has its objective anchored in academic research. Therefore, since its establishment in 2001, it has been conducted every 2 years aimed at collecting paramount data on several behaviors (e.g., PA and SB) of the European adults. The study protocol subscribed to the Declaration on Professional Ethics of the International Statistical Institute can be found here (http://www.europeansocialsurvey.org/about/ethics.html), and the ESS was approved by the ESS ERIC Research Ethics Committee. The legal bases used in European Social Survey are in accordance with the General Data Protection Regulation and national laws of each country involved in the study.

Probability sampling was applied in all countries to residents aged 15 years and older (excluding the homeless and the institutionalized population), comprising a total of 40,185 participants. The present study aimed at the adult population; therefore, participants under 18 were excluded from the analysis (*n* = 1,735). Participants who did not report data on PA, watching TV, depressive symptoms, and sociodemographic variables (*n* = 9,165) were excluded from the analysis. After considering inclusion criteria, the final sample consisted of 29,285 individuals (13,943 men; 15,342 women), with a mean age of 50.9 years (SD = 17.4) years. The mean age for men was 50.6 years (SD = 17.3) and 51.1 years (SD = 17.5) for women.

### Measures

#### TV Watching

Participants were asked “*how much time, in total, do you spent watching TV on an average weekday*.” The answers ranged from no time to >3 h/day, using intervals of 30 min. Based on a previous study (23), responses were recoded to “no time at all,” “ <1 h/day,” “2–3 h/day,” and “>3 h/day.”

#### Physical Activity

Information about PA was obtained with a single item asking: “*On how many of the last 7 days did you walk quickly, do sports, or other PA for 30 min or longer?*”. Even though PA was assessed with a single item, previous studies show that a single question is reliable (24). Individuals were grouped using computed information on PA, specifically: (1) ≤ 1 day/week; (2) 2–4 days/week; and (3) ≥5 days/week.

#### Depressive Symptoms

Participants completed the Center for Epidemiological Studies Depression Scale (CES-D8) as it measures depressive symptoms. This version contains eight questions (example: “*how much time they felt depressed in the last week*”). Each item is scored from 1 (none or almost none of the time) to 4 (all or almost all the time) and the score ranged from 0 to 24. Two items were reverse coded. A higher score indicates higher levels of depression symptoms. Specifically, a score of ≥9 was used as a cut-off value to identify elevated depression symptoms (25). The CES-D8 is a valid and reliable instrument for screening depression in the adult population in several countries (26).

#### Covariates

Sociodemographic variables such as age and sex were self-reported. Additionally, participants were asked how many people, including children, regularly lived in the household. Response options were dichotomised into lives with or without children in the household, and whether they lived with husband/wife/partner, and their correspondent legal status. Participants were asked what their occupation was in the past 7 days before the interview. Response options were used to create six categories: employed; student; retired; unemployed; sick or disabled; and housework, looking after children. To determine the living place, participants were asked to report whether they lived in a big city, suburbs, or outskirts of a big city, town or small city, country village, or home in the countryside. A new category named urban areas was created for those who answered that they lived in a big city, suburbs, or outskirts of a big city. Those who responded that they lived in a country village or home in the countryside were grouped into the rural area's category. Socioeconomic status was determined based on decile. Using the data, 1st−3rd, 4th−7th, and 8th−10th decile were grouped to create three groups: low, middle, and high socioeconomic status, respectively. Since the above mentioned sociodemographic variables are determinant factors of PA and SB (27) we selected them as covariates.

### Statistical Analysis

Descriptive statistics were calculated (means, standard deviation, and percentages) for the entire sample. The sample was grouped according to sex, considering PA, TV watching, and depression symptoms (28, 29). Student's *t*-test and Chi-square were used to compare variables such as socio-demographic characteristics, time spent TV watching, PA, and scores for depressive symptoms according to sex. To analyze the association between the time spent TV watching and scores for depressive symptoms, an ANCOVA was performed, adjusted for sex. Linear regression was conducted to analyze the association between PA and the score for depressive symptoms. First, an unadjusted model was performed. Further analyses were adjusted for employment status, living place, children, socioeconomic status, marital status, and age. Finally, according to PA frequency, a General Linear Model was used to analyze the effect of time spent TV watching on depressive symptoms. This analysis was stratified by sex and adjusted for employment status, living place, children, socioeconomic status, marital status, and age. Statistical analysis was performed using IBM SPSS Statistics v.25.0. The significance level was set to *p* < 0.05.

## Results

Descriptive statistics are presented in Table 1. The mean age of the participants was 50.9 years (SD = 17.4). Considering both sexes, the mean score for depressive symptoms was 5.3 (SD = 4.0) and 18.5% of the total sample had a score higher than 9. The mean score for men was 4.8 (SD = 3.7), whilst women had a mean score of 5.6 (SD = 4.2). More women than men reported a score for depressive symptoms higher than 9 (22.2 vs. 14.4%).

**Table 1:** Participants' characteristics stratified by sex in 2014.

	**Total**		**Men**		**Women**		***p***
	***n***	**% (CI 95%) or *M* (SD)**	***n***	**% (CI 95%) or *M* ± SD**	***n***	**% (CI 95%) or *M* ± SD**	
Age^a^		50.9 (17.4)		50.6 (17.3)		51.1 (17.5)	0.015
SES^b^							<0.001
Low	8,965	30.6 (29.7, 31.5)	3,680	26.4 (25.2, 27.4)	5,285	34.4 (33.2, 35.6)	
Medium	12,497	42.7 (41.8, 43.6)	6,144	44.1 (42.9, 45.3)	6,353	41.4 (40.2, 42.6)	
High	7,823	26.7 (25.8, 27.6)	4,119	29.5 (28.3, 30.7)	3,704	24.1 (22.9, 25.3)	
PA frequency (days/week)^a^		3.1 (2.6)		3.2 (2.6)		3.0 (2.6)	<0.001
PA frequency^b^							<0.001
0–1 days	10,183	34.8 (33.9, 35.7)	4,624	33.2 (31.8, 34.6)	5,559	36.2 (34.9, 37.5)	
2–4 days	9,725	33.2 (32.3, 34.1)	4,659	33.4 (32.0, 34.8)	5,066	33.0 (31.7, 34.3)	
≥5 days	9,377	32.0 (31.1, 32.9)	4,660	33.4 (32.0, 34.8)	4,717	30.7 (29.4, 32.0)	
TV watching (hours/day)^b^							<0.001
<1 h	7,252	24.8 (23.6, 26.0)	3,596	25.8 (24.1, 27.5)	3,656	23.8 (22.1, 25.5)	
1–2 h	9,063	30.9 (29.7, 32.1)	4,362	31.3 (29.6, 33.0)	4,701	30.6 (28.9, 32.2)	
2–3 h	7,442	25.4 (24.2, 26.6)	3,485	25.0 (23.3, 26.7)	3,957	25.8 (24.1, 27.5)	
>3 h	5,498	18.8 (17.6, 20.0)	2,488	17.8 (16.1, 19.5)	3,010	19.6 (17.9, 21.3)	
Living place^b^							0.004
Urban	18,864	64.4 (63.9, 64.9)	8,863	63.6 (62.8, 64.4)	10,001	65.2 (64.4, 66.0)	
Rural	10,421	35.6 (35.1, 36.1)	5,080	36.4 (35.6, 37.2)	5,341	34.8 (34.0, 35.6)	
Children^b^							<0.001
No	18,356	62.7 (62.1, 63.3)	9,135	65.5 (64.1, 66.3)	9,221	60.1 (59.3, 60.9)	
Yes	10,929	37.3 (36.7, 37.9)	4,808	34.5 (33.7, 35.3)	6,121	39.9 (39.1, 40.7)	
Marital status^b^							<0.001
Live with partner	18,371	62.7 (62.1, 63.3)	9,236	66.2 (65.4, 67.0)	9,135	59.5 (58.7, 60.3)	
Live without partner	10,914	37.3 (36.7,37.9)	4,707	33.8 (33.0, 34.6)	6,207	40.5 (39.7, 41.3)	
Score of depression (total)^a^		5.3 (4.0)		4.8 (3.7)		5.6 (4.2)	<0.001
Score of depression^b^							<0.001
0–9	23,872	81.5 (76.9, 86.1)	11,935	85.6 (79.5, 91.7)	11,937	77.8 (71.1, 84.5)	
>9	5,413	18.5 (13.9, 23.1)	2,008	14.4 (8.3, 20.5)	3,405	22.2 (15.5, 28.9)	
Employment status^b^							<0.001
Employed	15,702	53.6 (51.8, 55.4)	8,176	58.6 (56.5, 60.7)	7,526	49.1 (46.4, 51.8)	
Student	1,286	4.4(2.6, 6.2)	612	4.4 (2.3, 6.5)	674	4.4 (1.7, 7.1)	
Retired	7,761	26.5 (24.7, 28.3)	3,689	26.5 (24.4, 28.6)	4,072	26.5 (23.8, 29.2)	
Unemployed	1,634	5.6 (3.8, 7.4)	860	6.2 (4.1, 8.3)	774	5.0 (2.3, 7.7)	
Sick or disabled	841	2.9 (1.1, 4.7)	387	2.8 (0.7, 4.9)	454	3.0 (0.2, 5.7)	
Housework, looking after children	2,061	7.0 (5.2, 8.8)	219	1.6 (−0.4, 3.7)	1,842	12.0 (9.3, 14.7)	

*M, mean; SD, standard deviation; SES, socioeconomic status; PA, physical activity; TV, television*.

a *Tested with Student's t-test*.

b *Tested with chi-square test*.

The result of the ANCOVA is presented in Table 2. Specifically, the effect of time spent TV watching and the score for depressive symptoms, stratified by sex. Significant differences were found across all categories. The categories “1–2 h” and “>3 h” showed the lowest and the highest score of depressive symptoms in both sexes, respectively.

**Table 2:** Relationship between time spent watching TV and the score of depression in 2014.

	**Score of depression;** ***M*** **(SD)**			
	**Men**	***p***	**Women**	***p***
TV watching (hours/day)		<0.001^a^		<0.001^a^
<1 h	4.9 (0.1)		6.0 (0.1)	
1–2 h	4.6 (0.1)		5.8 (0.1)	
2–3 h	4.7 (0.1)		6.0 (0.1)	
>3 h	5.2 (0.1)		6.5 (0.1)	

*M, mean; SD, standard deviation; TV, television*.

*Analysis was adjusted for employment status, living place, children, socioeconomic status, marital status, age*.

a *Significant difference found between <1 and >3 h categories. Tested with ANCOVA and Bonferroni post-hoc test*.

The results of the linear regression model are presented in Table 3. Specifically, the parameters estimate of the relationship between PA and the score for depressive symptoms score are showed according to sex. Looking at the men sample, PA was negatively associated with the score for depressive symptoms in the unadjusted model (β = −0.17, 95% CI: −0.19, −0.15), and (β = −0.15, 95% CI: −0.18, −0.13) after adjusting to employment status, living place, children, socio-economic status, marital status, and age. Looking at the women sample, PA was negatively associated with depressive symptoms in the unadjusted model (β = −0.26, 95% CI: −0.29, −0.24), and (β = −0.23, 95% CI: −0.26, −0.21) after adjusting for the same sociodemographic variables reported in the adjusted model for men.

**Table 3:** Relationship between physical activity and score of depression in 2014.

	**β (95% CI) for the score of depression**			
	**Men**		**Women**	
	**Model 1**	**Model 2**	**Model 1**	**Model 2**
PA frequency	−0.17	−0.15	−0.26	−0.23
(days/week)	(−0.19, −0.15)	(−0.18, −0.13)	(−0.29, −0.24)	(−0.26, −0.21)

*CI, confidence interval; PA, physical activity*.

*Model 1: unadjusted*.

*Model 2: Adjusted for employment status, living place, children, socioeconomic status, marital status, and age*.

**Figure 1:**
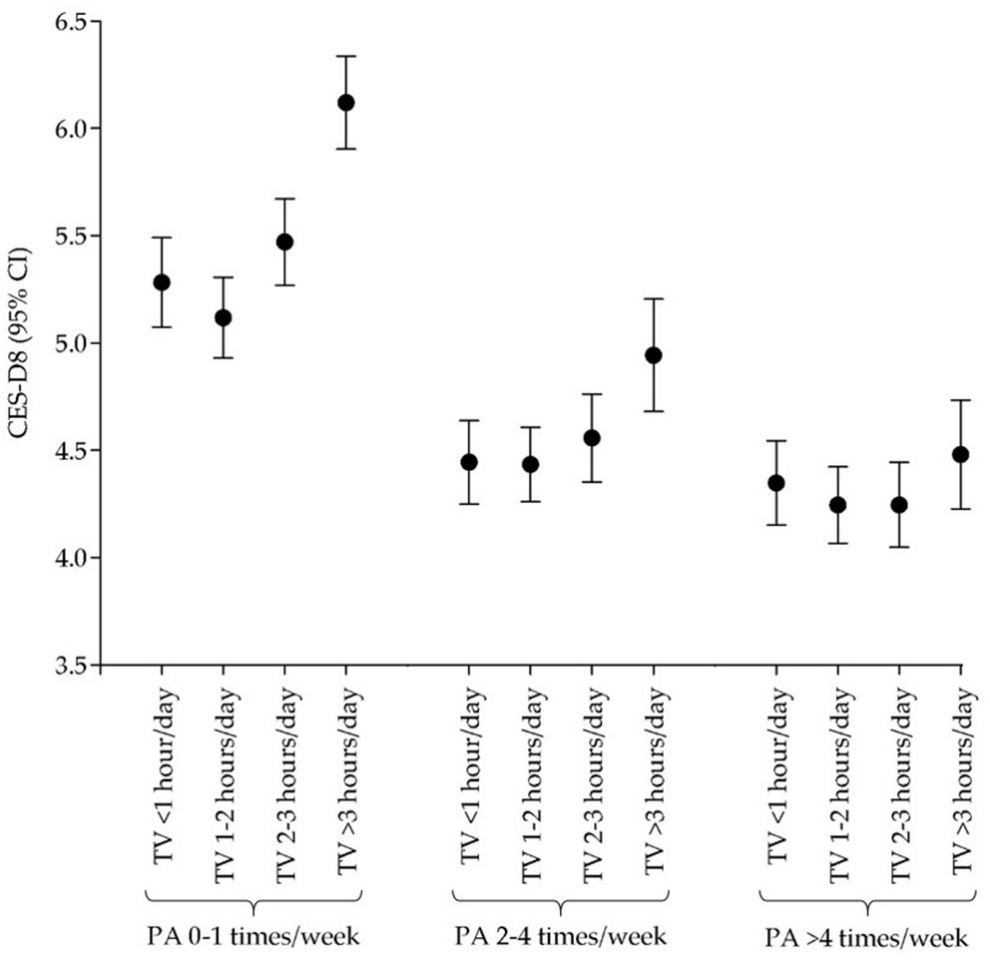
Relationship across physical activity, TV watching, and depressive symptoms in men.

**Figure 2:**
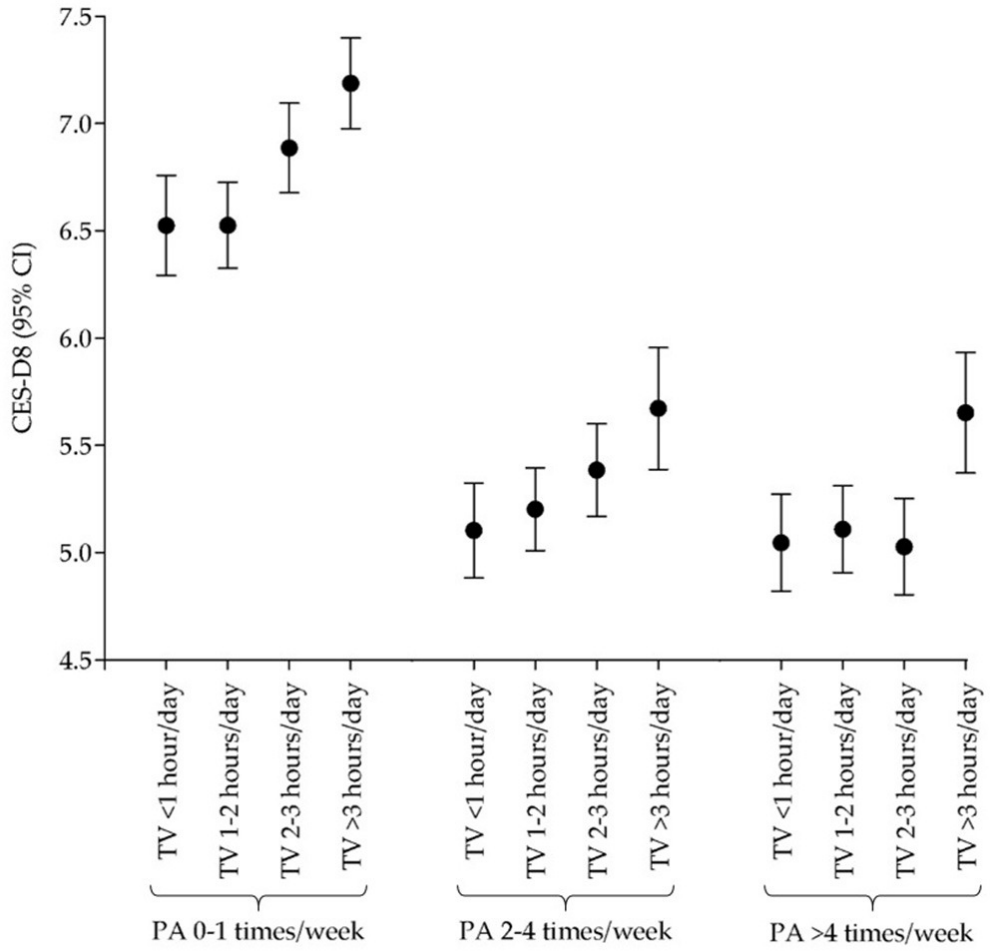
Relationship across physical activity, TV watching, and depressive symptoms in women.

The results of the general linear model analysis are displayed in Figure 1 (for men) and Figure 2 (for women). Both sexes who reported engaging 0–1 times per week in PA and spending >3 h/day watching TV had the highest score for depressive symptoms (men: 6.12, 95% CI: 5.90, 6.34; women: 7.92, 95% CI: 7.65, 8.19). Among men, those who reported engaging ≥5 days per week in PA and spending 1–2 h/day watching TV had the lowest CES-D8 score (4.25, 95% CI: 4.07, 4.42). While, among women, those who reported engaging 2–4 days per week in PA and spending <1 h/day watching TV had the lowest CES-D8 score (4.99, 95% CI: 4.73, 5.25). In each of PA frequency categories men and women who watched TV more than 3 ha day had the highest mean of CES-D8 score [men: (0–1 days/week), 6.12, 95% CI: 5.90, 6.34; (2–4 days/week), 4.94, 95% CI: 4.68, 5.20; (≥5 days/week), 4.48, 95% CI: 4.23, 4.73; women: (0–1 days/week), 7.92, 95% CI: 7.65, 8.19; (2–4 days/week), 5.94, 95% CI: 5.62, 6.27; (≥5 days/week), 6.43, 95% CI: 6.11, 6.75].

According to the current results, there is a significant pattern in which lower scores for depressive symptoms can be found in individuals with increased PA, independent of time spent TV watching. This tendency is more noticeable among those who engaged in 0–1 days/week vs. the other two categories. The same tendency can be observed when comparing sexes. These findings are suggestive of a moderating effect of PA in the increase of depressive symptoms due to the increased time spent TV watching.

## Discussion

This study aimed to analyze the independent association between time spent TV watching and depressive symptoms, as well as the relationship between PA participation and depressive symptoms, and investigate the potential moderating effect of PA in the relationship between TV watching and depressive symptoms in a large European adult population, controlling for sociodemographic variables. Our findings suggest that more time spent TV watching was associated with higher depressive symptoms. In contrast, increased levels of PA were associated with significant reduced depressive symptoms. Moreover, PA seems to show a moderating effect in the association between time spent TV watching and depressive symptoms.

Results showed that the association between TV watching and scores for depressive symptoms was higher in the independent models among individuals who spent >3 h/day watching TV. These results support the hypothesis that spending a greater amount of time TV watching is associated with increased depressive symptoms, providing further evidence on the detrimental effect of SB on mental health (14). Despite the uncertain mechanisms associating SB such as TV watching and depressive symptoms, existing literature points out potential factors such as lower social contact, poorer sleep quality (30), and also levels of metabolic markers suggesting a reduced insulin sensitivity and inflammation (31). Specifically, there is evidence that increased SB may be linked to reduced PA engagement, which can contribute to lower scores on mental health (14, 32). Nevertheless, previous research suggests that time spent TV watching is associated with increased ingestion of highly dense and nutritionally poor food (33), which is also related to increased inflammation (34), one factor that is related to depressive symptoms (35, 36).

Participants who spent <1 h/day TV watching had a higher score for depressive symptoms, regardless of PA engagement when compared to those who watched 1–2 h/day of TV and 2–3 h/day of TV (only in men). Accordingly, our results support previous research suggesting that individuals spending <1 h/day and more than 2 h/day watching TV have an increased depressive symptomatology among the adult population (37).

Previous investigations suggest that PA has a negative effect on depressive symptoms (38). In line with proposed hypothesis, our results indicate that PA may reduce depressive symptoms regardless of sex. However, women seem to benefit more from engaging in PA compared to men as a mean to reduce depressive symptoms. These results support evidence, suggesting a protective effect of PA against the possible development of depression (39).

In general, men had the lowest score for depressive symptoms when engaging in ≥5 times/week in PA. Among men engaging in ≥5 times/week in PA, those who spent 1–2 h/day TV watching had the lowest score of depression. For women, the lowest score of depressive symptoms was observed for those engaging 2–4 times/week in PA and reporting <1 h/day of TV watching. Bearing this in mind, our results support the findings of previous research suggesting that frequent engagement in PA reduces depression symptoms (8, 39). While the associations found in our study were substantial, the cross-sectional nature of the data prevents any interpretation of causality. Nevertheless, some plausible mechanisms of action could explain why PA reduces the harmful effect of watching TV in depression. Increased SB time has a negative effect on sleep (40). Depressed individuals have greater sleeping problems and lower PA levels (41). Research suggests that interleukin-6 (IL-6) and tumor necrosis factor (TNF-α) may be directly involved in sleep regulation (42). SB is associated with increased expression of those cytokines (31), on the other hand, PA can regulate the expression of those inflammatory biomarkers (43). PA has a positive effect on brain functioning throughout the lifespan (44). For instance, it was found that increased levels of plasmatic brain-derived neurotrophic factor (BDNF) after 4 sessions of 30-min cycling at an intensity of 60% of peak oxygen intake (45). BDNF has a key role in neurogenesis and has been negatively associated with sedentary time (44). Thus, we speculate that PA might attenuate the negative effect that SB time has on the expression of BDNF. Moreover, watching TV increases the levels of C-reactive protein (CRP) (22), which can be regulated through PA (46). Higher levels of CRP are associated with increased depression severity (47). Therefore, providing more opportunities for PA can be an effective strategy to prevent depression (9, 10).

### Implications of Findings

Current findings provide evidence on how PA may moderate the association between TV watching and depressive symptoms in the European adult. Future interventions should focus on the reduction of time spent TV watching and increasing PA participation as a mean to reduce depressive symptoms. Policies should promote less time TV watching in the early life stages and should be a priority since research has found that SB habits from earlier life years proceed to adulthood (48). Providing more opportunities for engaging in PA should be a priority for public authorities to reduce the individual and the economic burden associated with poor mental health (7).

### Strengths, Limitations, and Agenda for Future Research

The current study adds to the debate evidence about the association between SB and depressive symptoms, while moderating this relationship based on PA engagement. Additionally, this study addresses limitations related to previous studies (10) in which only simple association tests were conducted that do not account for the variability based on sociodemographic variables. The robustness in the statistical analysis, as well as the large sample size and controlling for sociodemographic variables are strengths of the current research.

Some limitations must be acknowledged. Study variables were self-reported, which are susceptible to bias. PA was obtained through a question, lacking data on frequency, intensity, time, and type based on the ACSM protocol (11). This lack of information prevents a more precise calculation of METs which are indicative on PA engagement. Thus, future studies should focus on collecting more data on PA engagement, considering its manifestations.

While this study controlled the associations between TV watching, PA, and depressive symptoms adjusted for employment status, living place, children, socioeconomic status, marital status, and age, future studies should consider other covariates such as body mass index, smoking, and alcohol consumption. Thus, current results should be considered based on covariates under analysis and caution must be taken when interpreting present evidence.

## Conclusions

The present study found that more time spent TV watching was associated with higher depressive symptoms. In contrast, more engagement in PA was negatively associated with depressive symptoms in European adults. Moreover, PA seems to moderate the association between the time spent TV watching and depression symptoms. Public health policies promoting mental health should include reducing time spent TV watching and the increase of PA as a mean to promote mental health among adults living in Europe.

## Data Availability Statement

The datasets presented in this study can be found in online repositories. The names of the repository/repositories and accession number(s) can be found in the article/supplementary material.

## Ethics Statement

The studies involving human participants were reviewed and approved by ESS ERIC Research Ethics Committee. The patients/participants provided their written informed consent to participate in this study.

## Author Contributions

JS, AM, and MP conceived and designed the study and performed the analysis and interpretation of the data. JS, AM, MP, AI, EG, GF, CD, FR, and AW drafted and approved the final version. JS, FR, and CD revised the document. All authors contributed to the article and approved the submitted version.

## Funding

This paper uses data from SHARE Wave 7 (doi: 10.6103/SHARE.w7.711). The SHARE data collection has been funded by the European Commission through FP5 (QLK6-CT-2001-00360), FP6 (SHARE-I3: RII-CT-2006-062193, COMPARE: CIT5-CT-2005-028857, SHARELIFE: CIT4-CT-2006-028812), FP7 (SHARE-PREP: GA N°211909, SHARE-LEAP: GA N°227822, SHARE M4: GA N°261982, DASISH: GA N°283646) and Horizon 2020 (SHARE-DEV3: GA N°676536, SHARE-COHESION: GA N°870628, SERISS: GA N°654221, SSHOC: GA N°823782) and by DG Employment, Social Affairs and Inclusion. Additional funding from the German Ministry of Education and Research, the Max Planck Society for the Advancement of Science, the U.S. National Institute on Aging (U01_AG09740-13S2, P01_AG005842, P01_AG08291, P30_AG12815, R21_AG025169, Y1-AG-4553-01, IAG_BSR06-11, OGHA_04-064, HHSN271201300071C) and from various national funding sources is gratefully acknowledged (see www.share-project.org). This project was supported by the Portuguese Foundation for Science and Technology, I.P., Grant/Award Number UID/CED/04748/2020.

## Conflict of Interest

The authors declare that the research was conducted in the absence of any commercial or financial relationships that could be construed as a potential conflict of interest.

## Publisher's Note

All claims expressed in this article are solely those of the authors and do not necessarily represent those of their affiliated organizations, or those of the publisher, the editors and the reviewers. Any product that may be evaluated in this article, or claim that may be made by its manufacturer, is not guaranteed or endorsed by the publisher.
